# The relationship between perceived social support and psychological resilience in Chinese adolescent Judo athletes: a cross-sectional study on the mediating role of depression and the moderating role of age

**DOI:** 10.3389/fpsyt.2025.1558351

**Published:** 2025-09-10

**Authors:** Wenjia Chen, Haozhe Wang, Zongyu Liu, Jiayi Yao, Dengshan Chu, Xueqiang Zhu, Haitao Niu

**Affiliations:** ^1^ School of Physical Education, China University of Mining and Technology, Xuzhou, China; ^2^ Zhejiang University, College of Education, Department of Sport & Exercise Science, Hangzhou, China; ^3^ School of Competitive Sport, Shandong Sport University, Jinan, Shandong, China; ^4^ School of Physical Education, Shandong University, Jinan, China

**Keywords:** adolescents, Judo athletes, perceived social support, depression, psychological resilience

## Abstract

**Introduction:**

Adolescent judo athletes face significant mental health risks due to high-intensity training and competition pressures. Perceived social support and psychological resilience are critical protective factors, yet their underlying mechanisms are not fully understood in this population. This study aimed to investigate the relationship between perceived social support and psychological resilience, examining the mediating role of depression and the moderating role of age.

**Methods:**

This cross-sectional study included 207 Chinese judo athletes (106 males, 101 females; mean age = 18.77 ± 2.57 years; age range = 14–33 years) recruited between July and October 2024. Participants completed the Perceived Social Support Scale (PSSS), the Center for Epidemiological Studies Depression Scale (CES-D), and the Sports Mental Toughness Questionnaire (SMTQ). Data were analyzed using descriptive statistics, Pearson correlation, and a moderated mediation model via the SPSS PROCESS macro.

**Results:**

Perceived social support was a significant positive predictor of psychological resilience (b=0.338,p<0.01). This relationship was fully mediated by depression (indirect effect = 0.163, 95% CI [0.099, 0.242]), which accounted for 56.79% of the total effect. Furthermore, age moderated the association between perceived social support and depression (interaction effect = 0.049, 95% CI [-0.165, 0.263]), indicating that the protective effect of social support against depressive symptoms weakened as athlete age increased.

**Discussion:**

The findings demonstrate that perceived social support enhances psychological resilience in adolescent judo athletes, largely by alleviating symptoms of depression. The moderating effect of age suggests that the efficacy of social support in buffering against depression may vary across different developmental stages. These results highlight the importance of fostering strong support systems for young athletes, with a potential need for age-tailored mental health interventions. Future research should explore these dynamics using longitudinal designs and in diverse athlete populations.

## 1 Introduction

Originating in Japan, Judo, as a high-intensity form of individual combat, places significant demands on athletes’ psychological well-being. Systematic reviews of Judo highlight the crucial role of psychological factors in this sport, emphasizing psychological attributes such as psychological resilience and self-confidence were key determinants of success for Judo athletes (1). Furthermore, research indicates that participation in Judo among children not only enhances physical fitness but also fosters psychological development, including improvements in discipline and self-control (2). Consequently, a thorough investigation of the factors influencing the psychological health and athletic performance of adolescent Judo athletes is of considerable importance for promoting their holistic development and optimizing their competitive outcomes.

Psychological resilience is recognized as a salient psychological attribute in athletic performance, referring to an individual’s capacity for positive adaptation in the face of adversity and stress (3). Individuals exhibiting higher psychological resilience have been found to be better equipped to regulate negative emotional states arising from stress, perceiving stressful situations as challenges rather than threats (4). In recent years, the concept of “sporting resilience” has gained increasing attention, further emphasizing the contribution of psychosocial social protective factors to athletes’ ability to cope with adverse circumstances (5). Given the need for Judo athletes to manage rigorous training regimens, competition-related pressure, weight management protocols, and potential injuries, psychological resilience is particularly important for them.

Social support typically refers to the various forms of support and assistance provided by an individual’ s social network system, such as care, attention, or respect from other members of the social network, which directly affects an individual’s health (6). Perceived social support is the subjective experience of individuals feeling support from others and society. It is distinct from objective social support, emphasized by an individual’s subjective judgment of available support (7). According to ecosystem theory, an individual’s development is the result of their continuous interaction with the environment (8). Individuals lacking perceived social support are more likely to experience negative emotions such as feelings of helplessness and depression (9). Conversely, support from coaches, teammates, friends, and family have helped athletes maintain psychological resilience and life balance (10). Previous research has found that perceived social support is an important distributional variable affecting psychological resilience (11).

Depression is a common negative emotional state that impairs an individual’s cognitive function (12) and moral judgment (13). Judo athletes are more prone to physiological problems such as muscle damage and decreased immunity (14), as well as mental health risks (15), due to weight control and high-intensity training (16). Psychological resilience, as a positive personality trait, effectively mitigates the negative effects of stress on individuals. Research has shown that there is a significant negative correlation between the level of psychological resilience and the level of depression in athletes (17). According to the buffering hypothesis of social support, perceived social support regulates the negative effects of stressful events on physical and mental health, and plays a protective role in maintaining mental health (7).

Furthermore, chronological age, as an important indicator of an individual’s physiological and psychological maturity, garner considerable scholarly attention in sport psychology research. Research has elucidated that athletes’ perceived social support needs vary across the lifespan (18), and that age exerts both direct and indirect effects on depression through feelings of loneliness, with depressive symptoms tending to escalate with advancing age (19). Adolescence represents a critical developmental juncture, characterized by a relatively heightened reliance on external sources of support. Research on inter-generational dynamics in Judo has underscored that Judo athletes spanning diverse age cohorts encounter disparate psychological challenges, necessitating the implementation of tailored support and intervention strategies (20). While extant research has probed psychological health concerns among Judo athletes, there remains a paucity of investigation into the collective influence of perceived social support, depression, and age on psychological resilience in adolescent Judo athletes. Grounded in the afore mentioned theoretical frameworks and empirical evidence (21, 22), this study posits the following three hypotheses:

Hypothesis 1: Perceived social support was positively associated with psychological resilience among Judo athletes.
Hypothesis 2: Depression mediates the relationship between perceived social support and psychological resilience among Judo athletes.
Hypothesis 3: Age positively moderates the relationship between perceived social support and depression among Judo athletes.

**Figure 1:**
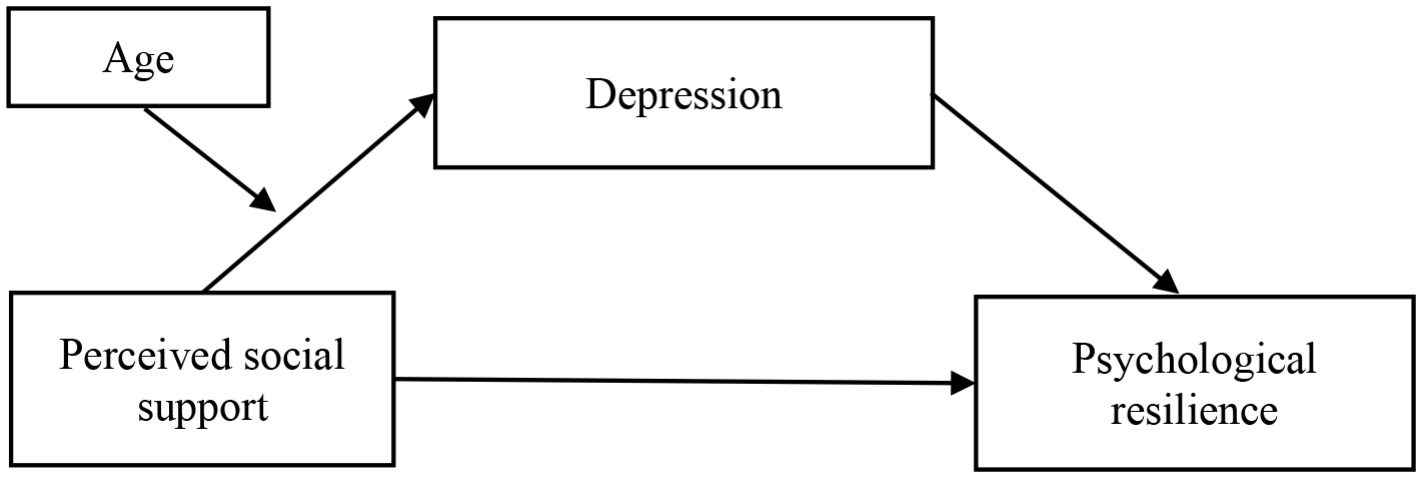
Research hypothesis model.

To summarize, this study has investigated Chinese adolescent judo athletes and examined the effects of factors such as perceived social support, depression, and age on mental toughness and their mechanisms of action, based on a review of related theories and literature and using a questionnaire survey method (Research framework is shown in **Figure 1**).

## 2 Participants and methods

### 2.1 Participants

This study employed a cross-sectional design and utilized a stratified cluster random sampling method to select 17 provinces from the 31 provincial-level administrative regions in China in July 2024. The selected provinces included Beijing, Shanghai, Tianjin, Chongqing, Shandong, Henan, Guangdong, Sichuan, Inner Mongolia, Shanxi, Shaanxi, Liaoning, Zhejiang, Jiangsu, Guangxi, Hubei, and Hunan. In China, judo athletes are divided into a clear hierarchy of competitive levels. This includes: Unranked (usually referred to as beginners or athletes who have not yet received an official rating), Level 2 Athletes (possessing a certain amount of provincial athletic ability at an intermediate level), Level 1 Athletes (at a level higher than Level 2 and approaching an advanced level), National Fitness Athletes (having reached a national level of athletic standards), and at the highest level, the International Fitness Athletes (representing an elite level of competitiveness in national or international competitions). These grades effectively reflect an athlete’s competitive ability and training background. Data were collected via an online questionnaire, with participants accessed the survey through a QR code link.

Inclusion criteria were: 1) members of a youth judo team; and 2) voluntary participation. Exclusion criteria were: 1) incomplete questionnaires; 2) judo athletes older than 25 years; and 3) individuals who did not provide informed consent. To ensure data quality, participants who completed the survey in under 300 seconds were excluded. Ultimately, 207 youth judo athletes were included in the analysis(106 males, 101 females and recruited between July and October 2024; mean age = 18.77 ± 2.57 years to aged between 14 and 33 years, with a mean age of 18.7 ± 2.5 years, encompassing late adolescents and young adults), with a response rate of 93.24%.

The sample size was determined based on several considerations: feasibility, as access to national-level adolescent judo athletes is limited, making a large-scale study challenging, with the final sample size reflecting the number of athletes available and willing to participate within the given time frame; and representative, as stratified cluster random sampling was used to select 17 provinces across China, aiming to obtain a geographically diverse sample that is reasonably representative of the population of adolescent judo athletes in China. To assess sample size adequacy, a *post-hoc* power analysis was conducted using G*Power 3.1.9.7 software. For a linear multiple regression model (fixed model, R² increase), based on a medium effect size (f² = 0.15), α = 0.05, with 2 tested predictors and 5 total predictors, the analysis showed that with our sample size (N = 207), the actual statistical power obtained was 0.9994, well above the conventional recommended threshold of 0.80. The corresponding critical F value was 3.04, with numerator degrees of freedom of 2 and denominator degrees of freedom of 201. This indicated that the current study had an adequate sample size to reliably detect medium effects.

All participants were informed of the study’s purpose and procedures before participating in the research. After completing the questionnaire, they were assured that they could withdraw at any time. Informed consent was obtained online from the researchers, ensuring compliance with ethical standards. Prior to data collection, all researchers received standardized training to ensure the survey process was consistently implemented. Throughout the study, researchers were available to address participants’ questions. The studies involving humans were approved by the Ethics Committee of Shandong University (Approval No. 2021-1-114).

### 2.2 Research instruments

(1) Center for Epidemiologic Studies Depression Scale (CES-D) This study used CES-D, developed by Radloff et al. in 1977 (23). The scale consisted of 20 items, with 16 items assessing negative effects and 4 items assessed positive effect. Responses were rated on a four-point scale: 0 for “rarely or none of the time,” and 3 for “most or all of the time”. The total score ranged from 0 to 60, with higher scores indicated more severe depressive symptoms. In this study, the internal consistency (Cronbach’s α) of the CES-D was 0.946.

(2) Sports Mental Toughness Questionnaire (SMTQ) This study also adopted SMTQ, developed by Sheard et al. in 2009 (24). The construct validation study of the Sports Mental Toughness Questionnaire (SMTQ) included 12 items assessing three dimensions: confidence, determination, and control. Responses were rated on a five-point Likert scale (1 = “strongly disagree,” 5 = “strongly agree”). The total score was obtained by summing the scores from all 12 items, with higher scores indicated greater mental toughness. The internal consistency (Cronbach’s 162 α) was 0.81 for confidence, 0.72 for determination, and 0.75 for control.

(3) Perceived Social Support Scale (PSSS) This study adopted PSSS compiled by Blumenthal JA et al (25). The scale contained 12 items. Items directly related to social support were divided into three dimensions according to the source of support: family, friends, and significant others, with each dimension comprising four items. This scale used a scoring system ranging from 1 (strongly disagree) to 7 (strongly agree). A higher total score indicated more perceived social support by the individual. The Cronbach’s α coefficient of this scale in this study was 0.935.

### 2.3 Statistical methods

All data analyses in this study were conducted using the Statistical Package for the Social Sciences (SPSS) version 26.0. First, Harman’s single-factor test was used to assess common method bias. Principal component analysis extracted 5 factors with eigenvalues greater than 1, with the first factor showing 25.53% of the variance (less than the 40% of the threshold), which indicated that common method bias was not a significant issue in this study.

Next, descriptive statistics and Pearson correlation analyses were performed for all variables. The mediating effect of depression and the moderating effect of age were then tested using Model 4 and Model 7, respectively, of the PROCESS macro. The Bootstrap method (with 5000 resamples) and 95% confidence intervals (CI) were used to evaluate the significance of the mediation and moderation effects. Effects were considered significant if the CI did not include 0. Before constructing the mediation model, all variables were standardized, and demographic factors such as education level and judo rank were included as covariates to control for potential confounding effects. There were no missing data in the 207 participant questionnaires, as all questions were answered completely.

## 3 Results

### 3.1 Descriptive statistics and correlation analysis

This study analyzed data from 207 Chinese adolescent judo athletes. Participants had a mean height of 174.208 ± 14.105 cm, an average weight of 79.614 ± 22.871 kg, and a mean BMI of 25.748 ± 6.097. The average age of the subjects was 18.744 ± 2.508 years. Regarding judo practice experience, 13 participants (6.2%) had practiced for less than 2 years, 85 (41.0%) for 2–5 years, 74 (35.7%) for 5–8 years, 24 (11.5%) for 8–10 years, and 11 (5.3%) for more than 10 years. In terms of education level, 17 participants (8.2%) had junior high school or lower education (typically ages 12–15 in China), 156 (75.3%) had high school education (typically ages 15–18 in China), and 34 (16.4%) had college or higher education (typically ages 18 and above in China). The distribution of current judo level was as follows: 53 participants (25.6%) had no official rank (meaning these athletes were beginners or had not yet obtained a formal rating in the Chinese Judo Association’s ranking system), 26 (12.5%) were second-level athletes, 85 (41.0%) were first-level athletes, 42 (20.2%) were national masters, and 1 (0.4%) was an international master (these ranks could be understood as: second-level athletes were at an intermediate level with certain provincial competitive ability; first-level athletes were above second-level, approaching an advanced level; national masters had reached a national competitive standard; and international masters indicated elite status in national or international competitions). Regarding acute sports injuries experienced in the past year, 74 participants (35.7%) reported having experienced injuries, while 133 (64.2%) had not. In terms of perceived stress in daily life, 29 participants (14%) reported high stress, 150 (72.4%) reported moderate stress, and 28 (13.5%) reported low stress. (Refer to **Table 1** for detailed demographic and training background characteristics).

**Table 1:** Demographic and training background characteristics of adolescent Judo athletes.

Characteristics	Category	Total (n=207)
Height (cm)		174.208 ± 14.105
Weight (kg)		79.614 ± 22.871
BMI		25.748 ± 6.097
Age (years)		18.744 ± 2.508
years of judo practice, n (%)	<2 years	13 (6.2%)
	2–5 years	85 (41.0%)
	5–8 years	74 (35.7%)
	8–10 years	24 (11.5%)
	>10 years	11 (5.3%)
Education level, n (%)	Junior high or below	17 (8.2%)
	High school	156 (75.3%)
	College or above	34 (16.4%)
Current judo level, n (%)	No rank	53 (25.6%)
	Second-level	26 (12.5%)
	First-level	85 (41.0%)
	National master	42 (20.2%)
	International master	1 (0.4%)
Experienced acute sports injury in the past year, n (%)	Yes	74 (35.7%)
	No	133 (64.2%)
Perceived stress in daily life, n (%)	High	29 (14%)
	Moderate	150 (72.4%)
	Low	28 (13.5%)

Data are described as n (%) or mean ± SD.

Descriptive statistics for all study variables are presented in **Table 2**. Among the 207 adolescent judo athletes, the mean total score for mental toughness (PR) was 45.70 (SD = 7.41), indicated a good level of mental toughness. The mean total score for depression (De) was 33.19 (SD = 7.64), with a mean score for negative affect (NA) of 27.29 (SD = 6.59) and for positive affect (PA) of 5.90 (SD = 1.93), reflected a moderate level of overall depression. The mean total score for perceived social support (PSS) was 66.19 (SD = 13.01). The scores for its dimensions, family support (FS) (M = 22.25, SD = 4.52), friend support (FrS) (M = 21.78, SD = 4.61), and significant other support (SOS) (M = 22.17, SD = 4.57), were similar, suggested that the adolescent judo athletes received balanced social support from various sources and that the overall level of support was relatively high. The standard deviations for these variables indicated a degree of individual variation within the sample.

**Table 2:** Descriptive statistics of variables and their dimensions.

Variable	N	Range	Minimum	Maximum	M	SE	SD	Variance
Depression (De)	207	40.00	20.00	60.00	33.19	0.5312	7.6430	58.416
Negative Affect (NA)	207	37.00	16.00	53.00	27.29	0.4577	6.5857	43.372
Positive Affect (PA)	207	8.00	4.00	12.00	5.90	0.1339	1.9269	3.713
Psychological Resilience (PR)	207	43.00	26.00	69.00	45.70	0.5151	7.4114	54.929
Perceived Social Support (PSS)	207	59.00	25.00	84.00	66.19	0.9040	13.0065	169.169
Family Support (FS)	207	23.00	5.00	28.00	22.25	0.3143	4.5220	20.449
Friend Support (FrS)	207	17.00	11.00	28.00	21.78	0.3206	4.6131	21.280
Significant Other Support (SOS)	207	22.00	6.00	28.00	22.17	0.3175	4.5687	20.873
Age	207	11.0	14.00	25.00	18.7729	0.17887	2.57351	6.623

**Figure 2:**
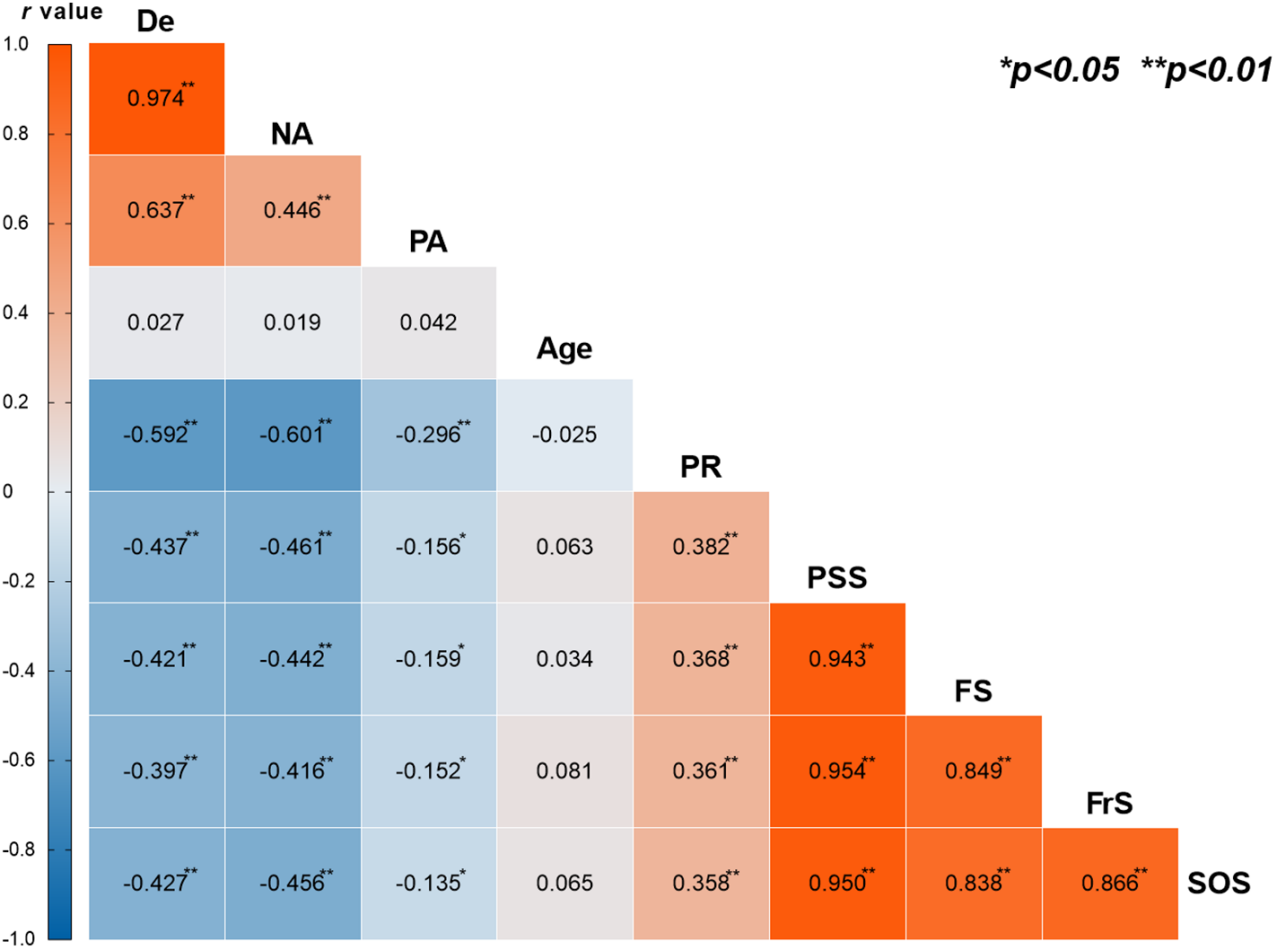
Pearson correlation coefficient result. Depression (De), Negative Affect (NA), Positive Affect (PA), Psychological Resilience (PR), Perceived Social Support (PSS), Family support (FS), Friend support (FrS), Significant others support (SOS). Correlation matrix showing relationships between variables. Blue, negative correlations; red, positive correlations. (*p<0.05, **p<0.01).

The results of the Pearson correlation analysis are shown in **Figure 2**. Among adolescent judo athletes, depression (De) was significantly negatively correlated with psychological resilience (PR) (r = -0.592, P < 0.01) and perceived social support total score (PSS) (r = -0.437, P < 0.01). Depression (De) also showed moderate negative correlation with the dimensions of perceived social support [family support (FS), friend support (FrS), significant other support (SOS)] (r range from -0.427 to -0.397, P < 0.01). Internally, depression (De) was very strongly positively correlated with its main component, negative affect (NA) (r = 0.974, P < 0.01), and significantly positively correlated with positive affect (PA) (r = 0.637, P < 0.01). Psychological resilience (PR) was significantly positively correlated with perceived social support total score (PSS) (r = 0.382, P < 0.01) and its dimensions (FS, FrS, SOS) (r range from 0.358 to 0.368, P < 0.01). Perceived social support total score (PSS) was highly positively correlated with its dimensions (FS, FrS, SOS) (r range from 0.943 to 0.950, P < 0.01), and these support dimensions were also highly correlated with each other (r range from 0.838 to 0.866, P < 0.01). In contrast, age, as a moderating variable, was not significantly correlated with depression (De; r = 0.027), psychological resilience (PR; r = -0.025), or perceived social support (PSS; r = 0.063) (all P > 0.05). The correlations between the variables were consistent with theoretical expectations, provided an empirical basis for the subsequent in-depth analysis of mediation and moderation effects.

### 3.2 Tests of the mediating effect of depression

The PROCESS macro Model 4 was used to test the mediating effect of depression on the relationship between perceived social support and psychological resilience. After controlling for demographic variables, the results (**Table 3**) showed that 1) perceived social support had a significant positive predictive effect on psychological resilience (B = 0.287, p<0.01); 2) perceived social support had a significant negative predictive effect on depression (B=-0.392, p<0.01); 3) after including depression, the predictive effect of perceived social support on psychological resilience weakened but remained significant (B = 0.124), and depression had a significant negative predictive effect on psychological resilience (B=-0.417, p<0.01).

**Table 3:** Test table for the mediating effect model of depression.

Independent variables	Model 1 (Psychological resilience)			Model 2 (Depression)			Model 3 (Psychological resilience)		
	*B*	*t*	*β*	*B*	*t*	*β*	*B*	*t*	*β*
Height (cm)	0.005	0.886	0.064	0.002	0.430	0.028	0.005	1.126	0.076
Weight (kg)	-0.000	-0.083	-0.006	0.000	0.147	0.010	-0.000	-0.030	-0.002
Is there any chronic sports injury	-0.008	-0.054	-0.004	-0.234	-1.692	-0.114	-0.106	-0.734	-0.052
Perceived stress in daily life	0.287*	2.138	0.151	-0.449**	-3.717	-0.236	0.100	0.777	0.053
Years of practicing judo	-0.034	-0.463	-0.032	-0.014	-0.215	-0.013	-0.040	-0.585	-0.038
Training frequency per week	0.140	1.747	0.120	-0.099	-1.380	-0.085	0.098	1.318	0.084
Sports injury in the past year	0.101	0.654	0.049	-0.237	-1.704	-0.114	0.003	0.018	0.001
Training duration per session	0.086	0.565	0.040	0.138	1.011	0.065	0.143	1.012	0.067
Perceived social support	0.287**	4.089	0.287	-0.392**	-6.217	-0.392	0.124	1.733	0.124
Depression							-0.417**	-5.652	-0.417
*R* 2	0.166			0.327			0.282		
*F*	*F*(9,197)=4.342,*p* = 0.000			*F*(9,197)=10.624,*p* = 0.000			*F*(10,196)=7.716,*p* = 0.000		

*p<0.05 **p<0.01; B, unstandardized regression coefficient; β, standardized regression coefficient; SE, standard error; t, t-test statistic; CI, confidence interval.

Further combining the Bootstrap results (**Table 4**), it can be seen that the indirect effect of perceived social support on psychological resilience through depression was significant (β=0.163, 95%CI=[0.099, 0.242]), accounting for 56.79% of the total effect, and the direct effect was 0.124, accounting for 43.21% of the total effect. In summary, depression played a complete mediating role in the process of perceived social support affecting psychological resilience, supporting hypothesis H2. The confidence intervals of the total mediating effect and indirect effect did not include 0 and were significant at the 0.05 level.

**Table 4:** Mediating effects of depression between perceived social support and psychological resilience.

Effect types	Path	95%CI	Effect
Direct effect	Perceived social support=>Psychological resilience	[-0.016~ 0.264]	0.124
Indirect effect	Perceived social suppor=>Depression=>Psychological resilience	[0.099 ~ 0.242]	0.163	
Total effect	Perceived social suppor=>Psychological resilience	[0.150 ~0.425]	0.287**

(*p<0.05, **p<0.01).

### 3.3 Testing the moderating effect of age

The moderated mediation model was further tested using Model 7 of the PROCESS macro. After controlling for demographic variables, the results (**Table 5**) showed that the interaction term between perceived social support and age had a significant negative predictive effect on depression (β=-0.144, p<0.05), while the interaction term between depression and age had no significant predictive effect on psychological resilience (β=-0.035, p>0.05). This indicated that age significantly moderated the direct relationship between perceived social support and depression but did not moderate the relationship between psychological resilience and depression.

**Table 5:** Mediation model test table with moderation.

Variables	Psychological resilience			Depression		
	β	t	p	β	t	p
Perceived social support	0.117	1.592	0.113	-0.375	-5.978	0.000**
Age	-0.010	-0.115	0.908	-0.070	-0.855	0.394
Perceived social support*Age	-0.063	-0.858	0.392	-0.144	-2.408	0.017*
Height (cm)	0.005	1.129	0.260	0.002	0.422	0.673
Training duration per session	0.153	1.064	0.289	0.146	1.079	0.282
Weight (kg)	0.000	0.046	0.963	0.001	0.348	0.728
Training frequency per week	0.099	1.307	0.193	-0.109	-1.528	0.128
Experienced acute sports injury in the past year	-0.007	-0.048	0.962	-0.240	-1.745	0.083
Years of practicing judo	-0.034	-0.374	0.709	0.035	0.407	0.685
Perceived stress in daily life	0.111	0.843	0.400	-0.405	-3.334	0.001**
Is there any chronic sports injury	-0.116	-0.789	0.431	-0.247	-1.790	0.075
Depression	-0.426	-5.647	0.000**			
Depression*Age	-0.035	-0.461	0.645			
R^2^	0.285			0.348		
F	F (13,193)=5.924,p=0.000			F (11,195)=9.481,p=0.000		

*p<0.05 **p<0.01; β, standardized regression coefficient; t, t-test statistic.

**Figure 3:**
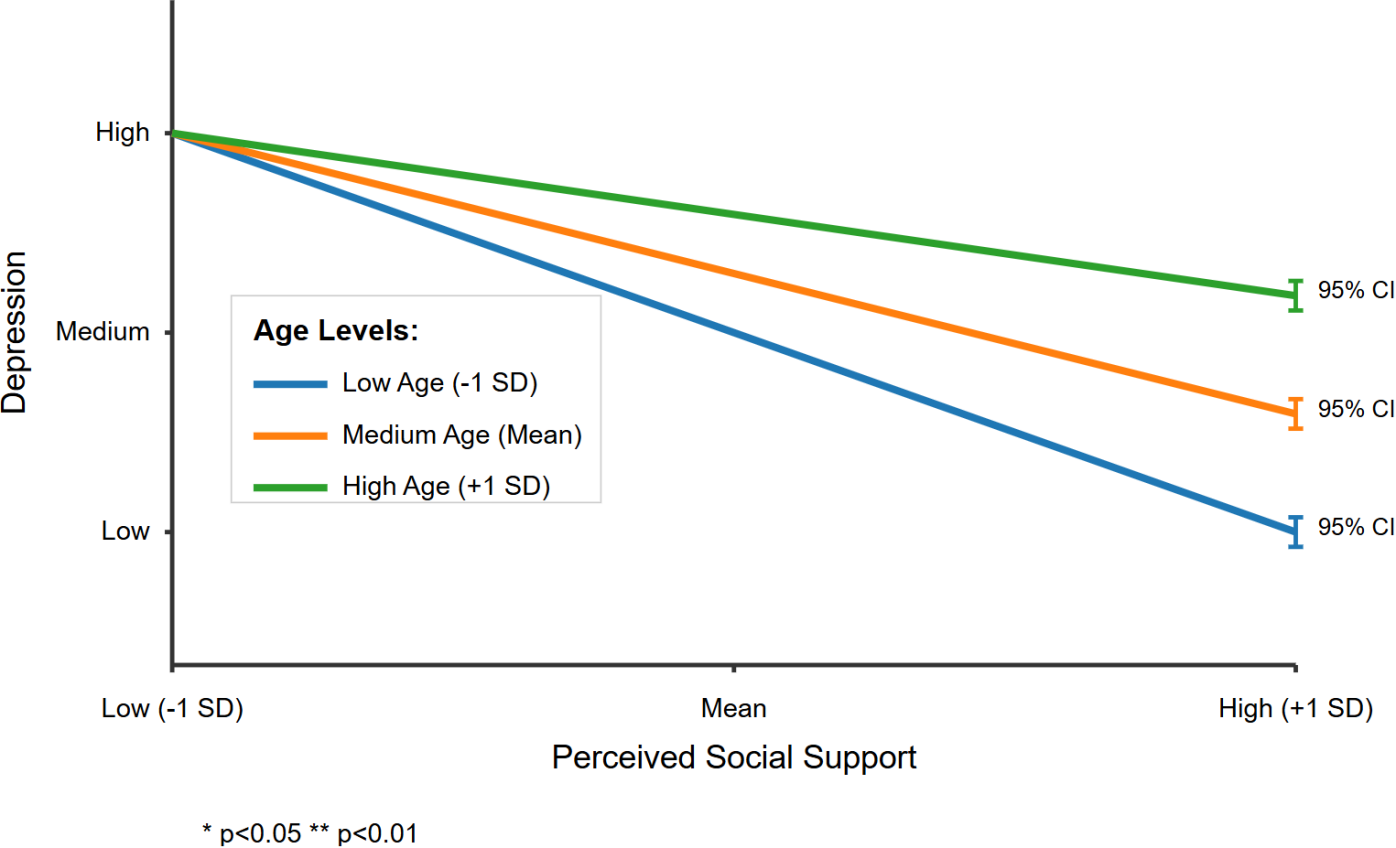
The moderating effect of age on the relationship between perceived social support and depression. (*p<0.05, **p<0.01).

To facilitate the interpretation of the moderating effect of age, the conditional direct effects of perceived social support on depression were examined at three age levels: low (-1SD), medium (M), and high (+1SD) (**Table 6** and **Figure 3**). The results revealed that as age increased from low to high, the direct effect of perceived social support on depression gradually weakened: from significant (Effect=0.181, SE = 0.099, 95% CI=[-0.014, 0.375], p<0.05) to medium (Effect=0.117, SE = 0.074, 95% CI=[-0.027, 0.262]), and then to non-significant (Effect=0.054, SE = 0.109, 95% CI=[-0.159, 0.268]).

**Table 6:** Conditional direct effect results.

Level	Level value	Effect	SE	t	LLCI	ULCI
Low level (-1SD)	-1.000	0.181	0.099	1.820	-0.014	0.375
Mean	-0.000	0.117	0.074	1.592	-0.027	0.262
High level (+1SD)	1.000	0.054	0.109	0.497	-0.159	0.268

LLCI represents the lower limit of the 95% confidence interval for the estimate, and ULCI represents the upper limit of the 95% confidence interval for the estimate.

The moderated mediation analysis for Model 59 also examined the conditional indirect effects of perceived social support on depression through basic psychological needs at different age levels (**Table 7**). For depression as a mediator, when at a low level, CI=[0.023, 0.199], Effect=0.090; when at the mean level, CI=[0.095, 0.242], Effect=0.160; when at a high level, CI=[0.126, 0.384], Effect=0.239. At low, medium, and high levels, the Bootstrap 95% CI did not include 0, indicating a mediating effect. The above analysis suggested that the mediating effect was consistent across different levels, indicated that there might not have been a moderated mediation effect.

**Table 7:** Conditional indirect effect results.

Mediator	Level	Level value	Effect	BootSE	BootLLCI	BootULCI
Depression	Low level (-1SD)	-1.000	0.090	0.043	0.023	0.199
	Mean	0.000	0.160	0.038	0.095	0.242
	High level (+1SD)	1.000	0.239	0.065	0.126	0.384

## 4 Discussion

### 4.1 The relationship between perceived social support and mental toughness in adolescent judoka

This study revealed a significant positive correlation between perceived social support and psychological resilience, consistent with previous research findings (26). This validation of Hypothesis H1 could be interpreted through two distinct mechanisms: Firstly, social support functioned as a provider of psychological resources (27). As a primary mode of influence within the interpersonal domain, social support significantly affected the health behaviors of adolescent athletes (28). The stress-buffering model suggests that enhanced social support mitigates the negative effects of stress and improves quality of life (29). When young Judo athletes perceived strong social support, they exhibited greater confidence and resilience in the face of training and competition challenges. Diverse sources of social support—including family encouragement, coaching guidance, and peer support—helped athletes recognize that they were not isolated when confronting difficulties, thereby enhancing their psychological resilience. Secondly, social support played a role in regulating negative emotions (30). Perceived social support provided athletes with emotional comfort and a sense of security (31), which was particularly valuable in the high-intensity environment of Judo training and competition. It was argued that adolescent athletes with robust support networks were more effective in perceiving and utilizing available psychological and material resources when managing setbacks and stress (32). This enhanced capacity for resource utilization improved their quality of life and strengthened their ability to leverage social resources, consequently reducing the impact of negative emotions such as loneliness, depression, and anxiety on psychological health development.

A large body of research supports the positive predictive role of perceived social support on psychological resilience. Lu et al.’s study of 218 university student-athletes found a significant positive relationship between coach social support and athletes’ psychological resilience (33), with coach support moderating the stress-burnout relationship in athletes. Sarkar et al.’s systematic review indicated that a strong social support network was one of the key protective factors for student-athletes in coping with stress and enhancing psychological resilience (34). These studies, from various perspectives, validated the positive influence of perceived social support on enhancing athletes’ psychological resilience. Young Judo student-athletes frequently face physical and mental stress from high-intensity training and competition. Due to their young age and immature psychological development, they are particularly vulnerable to psychological problems. Therefore, there was an urgent need to implement effective measures to enhance the social support of young Judo athletes to strengthen their psychological resilience.

### 4.2 The mediating effect of depression

The e results from this study demonstrated a significant negative correlation between perceived social support and depression. According to the main effects model, perceived social support positively influences individuals’ psychological well-being by enhanced coping abilities, reduces the appraisal of problem severity, and mitigates the adverse effects of stress, including depression and anxiety (35). Athletes with high perceived social support exhibited greater confidence and resilience when facing challenges, positively influencing depressive symptoms. Furthermore, social support networks provided multifaceted assistance through emotional, instrumental, informational, and appraisal support (36), thereby alleviating athletes’ psychological stress and lowering the risk of depression. Social support acted both as a direct protective factor against stress-related harm and as a buffer against the negative impact of stressors on health (37). Notably, support from family members, coaches, and teammates played a crucial role in maintaining athletes’ psychological health (38).This study found a significant negative correlation between psychological resilience and depression, confirming its predictive value for the psychological health status of adolescent Judo athletes. These findings align with previous research indicating that enhanced psychological resilience was associated with reduced levels of anxiety and depression (39). Individuals demonstrating high psychological resilience exhibited superior adaptability and psychological flexibility when confronting adversity, enabling them to manage stress more effectively, lowering the risk of depression (40). Moreover, corroborated prior findings demonstrate that psychological resilience moderated the relationship between stressors and depression (41). Consequently, enhanced psychological resilience in adolescent athletes held significant value for depression prevention and alleviation.

Conservation of Resources (COR) theory posits that both external resources (sports activities) and internal resources (psychological attributes) mitigate life and training-related stress (42), thereby improving athletic performance and competitive outcome. Sustaining athletes’ training motivation and performance during prolonged, high-intensity training periods necessitated organizational-level support of external resources, including the implementation of motivation-oriented and health-conscious training principles, the enhancement of social support systems, and the cultivation of a positive training environment. Concurrently, it was essential to focus on developing athletes’ internal resources, particularly psychological resilience and self-efficacy (43).In conclusion, perceived social support enhanced psychological resilience in adolescent Judo athletes by reduced depression, playing a crucial role in their overall physical and mental development. The study results suggested that consistently enhanced perceived social support in adolescent Judo athletes led to decreased depression levels, promoted improved emotional regulation and enhanced psychological resilience, thus confirming Hypothesis H2. These insights hold significant implications for the development of psychological health interventions for adolescent Judo athletes.

### 4.3 The moderating role of age

This study found that age significantly moderated the direct relationship between perceived social support and depression in adolescent Judo athletes. This finding aligns with previous research (44, 45). These findings could be explained from three perspectives, Firstly, Adolescent athletes were at a critical developmental period where external support was essential for psychological well-being. Young athletes who felt supported were more likely to develop positive self-identity and coping strategies, reducing the risk of depression (46). In contrast, with increasing age, individuals adopted negative coping mechanisms, hindering the effective utilization of social support (47).Secondly, young professional athletes typically lived in collective environments where basic material needs could be met. As age increased, needs became more diversified (48). School staff promptly identified young athletes’ problems and provided support; older athletes faced different sources of stress. At the same time, older athletes developed more diverse coping mechanisms, relying not only on social support but also on internal resources and self-regulation. Young athletes prioritized emotional support when establishing social networks (49), while older individuals placed greater emphasis on practical problem-solving and pragmatic advice. Thirdly, with increasing age, the proportion of family members in one’s social network increased, and this support did not always meet specific needs (50). At the same time, the proportion of social partners such as friends decreased, leading to a reduction in emotional support and social interaction, which consequently exacerbated the likelihood of depression. Inter-generational research on Judo emphasized that Judo coaches played a vital role in bridging the gap between participants of different age groups, which was consistent with our research findings on changes in social networks (20).

The present study found that gender did not significantly influence the relationship between perceived social support and mental toughness in judo athletes, which was inconsistent with the results of some studies of adolescent athletes (51). Other studies suggested that gender influenced the extent to which athletes benefited from social support (52). These inconsistencies may stem from the type of sport in the sample, age, or differences in the gender composition of participants in previous studies. Furthermore, within the context of China’s collectivist culture, the role and expression of social support differed significantly from Western individualistic cultures (53). Yamakawa et al. found that the traditional teacher-student relationship played a unique role in providing social support (54), a relationship that was less prominent in Western Judo training environments. Therefore, replicating this study in other cultural contexts would help to validate the universality of our research findings. In conclusion, age was an important moderated factor in the prevention and intervention of psychological health in young Judo athletes. Athletes of different ages had different psychological characteristics and required targeted measures to effectively safeguard their physical and mental health.

### 4.4 Implementation strategies for psychological health interventions in adolescent Judo athletes

The findings of this study provide a theoretical basis for psychological health interventions in adolescent Judo athletes. The implementation of these interventions emphasized age-specific considerations. For younger athletes (12–15 years), an established a multi-tiered support network comprising family, coaches, and peers would be crucial, whereas older athletes (16–19 years) require more specialized problem-solving skills and cheer planning counseling. An early identification mechanism for depressive symptoms would be realized through a quarterly psychological health screening system, coupled with training for coaches in recognized signs of depression. Furthermore, cognitive behavioral therapy and mindfulness-based stress reduction techniques would be integrated into daily training. Enhanced psychological resilience would be achieved through “adversity simulation training” to strengthen coping abilities in the face of setbacks, combined with experience sharing from elite athletes and a systematic resilience development curriculum. The establishment of a multidisciplinary support team, the development of individualized psychological development pathways, the provision of readily accessible psychological health resource platforms, and a periodic evaluation and optimization mechanism ensured the effective implementation of intervention strategies and comprehensively promote the psychological well-being and athletic performance of adolescent Judo athletes.

## 5 Conclusion

This study explores the relationships among perceived social support, depression, and psychological resilience in Chinese adolescent Judo athletes. The results revealed that perceived social support was significantly positively correlated with psychological resilience; depression played a complete mediating role in this relationship; and age significantly moderated the relationship between perceived social support and depression, with this association weakening as age increased. These findings suggest that constructing diversified social support networks and implemented age-differentiated intervention strategies are significant importance for enhancing the psychological health of adolescent Judo athletes. Younger athletes, in particular, require strengthened social support to mitigate the risk of depression. Future research could focus on: 1) employing longitudinal study designs to verify the causal relationships among variables; 2) expanding the study population to athletes from different cultural backgrounds and competitive levels; 3) validating the effectiveness of differentiated intervention programs; 4) integrating physiological indicators to explore the biological underpinnings of psychological resilience; 5) comparing psychological health patterns across different athletic disciplines; and 6) utilizing mixed-methods research to gain an in-depth understanding of the social support needs of athletes at different ages.

## Data Availability

The original contributions presented in the study are included in the article/Supplementary Material. Further inquiries can be directed to the corresponding author.
